# Leptin and Nutrition in Gestational Diabetes

**DOI:** 10.3390/nu12071970

**Published:** 2020-07-02

**Authors:** Antonio Pérez-Pérez, Teresa Vilariño-García, Pilar Guadix, José L. Dueñas, Víctor Sánchez-Margalet

**Affiliations:** 1 Department of Medical Biochemistry and Molecular Biology, and Immnology, School of Medicine, Virgen Macarena University Hospital, 41009 Seville, Spain; tvgarcia@gmail.com; 2 Obstetrics and Gynecology Service, Virgen Macarena University Hospital, 41009 Seville, Spain; pilarguadix@gmail.com (P.G.); jlduenas@us.es (J.L.D.)

**Keywords:** nutrition, polyphenolic compounds, bioactive compounds, leptin resistance, obesity, inflammation, gestational diabetes mellitus, Mediterranean diet

## Abstract

Leptin is highly expressed in the placenta, mainly by trophoblastic cells, where it has an important autocrine trophic effect. Moreover, increased leptin levels are found in the most frequent pathology of pregnancy: gestational diabetes, where leptin may mediate the increased size of the placenta and the fetus, which becomes macrosomic. In fact, leptin mediates the increased protein synthesis, as observed in trophoblasts from gestational diabetic subjects. In addition, leptin seems to facilitate nutrients transport to the fetus in gestational diabetes by increasing the expression of the glycerol transporter aquaporin-9. The high plasma leptin levels found in gestational diabetes may be potentiated by leptin resistance at a central level, and obesity-associated inflammation plays a role in this leptin resistance. Therefore, the importance of anti-inflammatory nutrients to modify the pathology of pregnancy is clear. In fact, nutritional intervention is the first-line approach for the treatment of gestational diabetes mellitus. However, more nutritional intervention studies with nutraceuticals, such as polyphenols or polyunsaturated fatty acids, or nutritional supplementation with micronutrients or probiotics in pregnant women, are needed in order to achieve a high level of evidence. In this context, the Mediterranean diet has been recently found to reduce the risk of gestational diabetes in a multicenter randomized trial. This review will focus on the impact of maternal obesity on placental inflammation and nutrients transport, considering the mechanisms by which leptin may influence maternal and fetal health in this setting, as well as its role in pregnancy pathologies.

## 1. Introduction

Gestational diabetes (GDM) is a hyperglycemic state that is recognized for the first time during pregnancy [1], and its pathophysiology is not fully clarified yet. GDM is one of the most common complications in pregnancy, affecting 3–8% of all pregnancies [2]. This prevalence has increased in recent decades (≥20% of pregnancies in some parts of the world), both in developed and developing countries, due to increased average age of pregnant women and increased obesity [3], one of the greatest public health challenges of the 21st century. Although the GDM phenotype is highly heterogeneous [4], half of its prevalence can be explained by overweight and obesity [5]. Indeed, obese women have an increased risk of GDM compared to women of normal weight [4]. Moreover, in women with GDM, pre-pregnancy obesity, excessive gestational weight gain and poor glycemic control are also linked with others pregnancy complications, such as gestational hypertension and preeclampsia [6]. It has even been reported that excessive gestational weight gain was the variable with the greatest effect on the probability of a newborn with macrosomia (a conditions associated with an increased risk of perinatal mortality and neonatal morbidity). GDM also increases, in turn, the risk, in both mother and offspring, of developing type 2 diabetes, metabolic syndrome and obesity [7,8]. Therefore, obesity during pregnancy is an important risk factor for adverse health outcomes both in the mother and offspring, and imposes substantial economic burdens. Therefore, prevention of obesity would be directly related to a lower risk of GDM. Impaired glucose homeostasis in GDM is also related to higher production of reactive oxygen species (ROS), consequently depleting the anti-oxidative status. This is why, to restrain the spread of epidemic excess weight, women must receive a comprehensive intervention before, during, and after pregnancy. Lifestyle changes, including nutrition initiated during early pregnancy, have been unsuccessful overall in preventing GDM in at-risk obese women [9]. Now, there is a consensus regarding the need for effective interventions targeting obesity and lifestyle that reduce the metabolic burden earlier in life, well before motherhood, and it underlines that such interventions may benefit both the mother and the future offspring. Although energy restriction leading to weight loss is a successful dietary intervention for improving obesity-associated metabolic disorders, other dietary interventions, such as those leading to a reduction in adipose tissue inflammation regardless of weight loss, have not been explored in detail. In this sense, leptin, produced by adipocytes, is a key regulator of appetite and is present in elevated concentrations in obesity [10]. Therefore, new research into nutritional mechanisms that restore leptin metabolism and signals of energy homeostasis may inspire new treatment options for obesity-related disorders such as GDM. In this review, we wanted to address the current insights and emerging concepts on potentially valuable nutrients and food components to modulate leptin metabolism. Moreover, obesity is associated with a chronic low-grade inflammation in the adipose tissue [11], and several dietary food components, such as phenols, peptides, and vitamins, are able to decrease the grade of inflammation and improve leptin sensitivity by up- or down-regulation of leptin-related genes. Others food components, such as saturated fatty acids should be avoided, since they may worsen chronic inflammation, subsequently increasing the risk for pathological complications. Finally, given the crucial role that the placenta plays in mediating pregnancy outcomes, it is important to consider the impact of micronutrient supplementation on the mechanisms associated with placental function, as well as maternal and fetal homeostasis.

## 2. Leptin

The hormone leptin, discovered in 1994 [12], critically regulates body weight and metabolism at central level in the brain [13], and disruption of leptin/leptin receptor (LEPR) signaling results in morbid obesity and severe metabolic disease [14,15]. In individuals of normal weight, the brain responds to increased plasma leptin levels by reducing food intake and increasing energy expenditure [16,17]. Leptin and leptin receptors are highly expressed in the preoptic area (POA), in the arcuate nucleus (ARC) of the hypothalamus as well as in other regions, such as the lateral hypothalamus, ventromedial hypothalamus and dorsomedial hypothalamus (DMH) [18]. There, it regulates energy homoeostasis and the neuroendocrine function, among other functions [19]. In these regions, leptin signaling is mediated by the JAK2/Stat3 pathway, in which several negative regulators of JAK2, including SOCS3 and PTP1B, have been reported to promote obesity [20,21], supporting the notion that JAK2 inhibitory molecules increase risk for leptin resistance and obesity. Therefore, hyperleptinemia and hypothalamic inflammation in diet-induced obesity may activate a common negative regulator of leptin signaling, SOCS3 or PTP1B, and contribute to central leptin resistance. In fact, up-regulation of SOCS3 in proopiomelanocortin (POMC) neurons leads to impairment of STAT3 signaling, with consequential leptin resistance and obesity, as well as glucose intolerance [22]. It has also been reported that mice with whole body or neuron-specific deletion of PTP1B are hypersensitive to leptin, and are resistant to diet-induced obesity [23]. Importantly, obesity is associated with impaired adipose sympathetic nerve transmissions [24,25], but the underlying mechanism is poorly understood. In this context, the leptin resistance at a central level may prevent negative feedback on the anti-inflammatory action of the sympathetic nervous system (SNS) [15,26]. That is why leptin is now considered one of the adipokines responsible for the inflammatory state found in obesity that could predispose to GDM. Surprisingly, Sh2b1 (an SH2 and PH domain-containing adaptor protein) [27,28] has emerged as an endogenous sensitizer for leptin action on the sympathetic nervous system (SNS) and energy expenditure, perhaps by enhancing JAK2 activation [29]. In this way, the LepR Sh2b1 neuron mediates leptin stimulation of the SNS and supports the preservation of adipose SNS against degeneration [30].

Apart from the JAK-2/Stat-3 pathway, activation of the MC4R signaling pathway by proopiomelanocortin (POMC)-derived melanocyte stimulating hormone (MSH) peptides also represents a critical convergence point in the control of body weight. The leptin–melanocortin pathway (MC4R pathway) integrates parallel inputs from the orexigenic peptides ghrelin, neuropeptide Y (NPY) and agouti-related peptide (AgRP), and activation of the MC4R pathway dominantly counteracts these orexigens. Limited efficacy of lifestyle intervention in individuals with mutations in gene-encoding components of this pathway demonstrates its importance in the control of body weight homeostasis [31,32].

**Figure 1:**
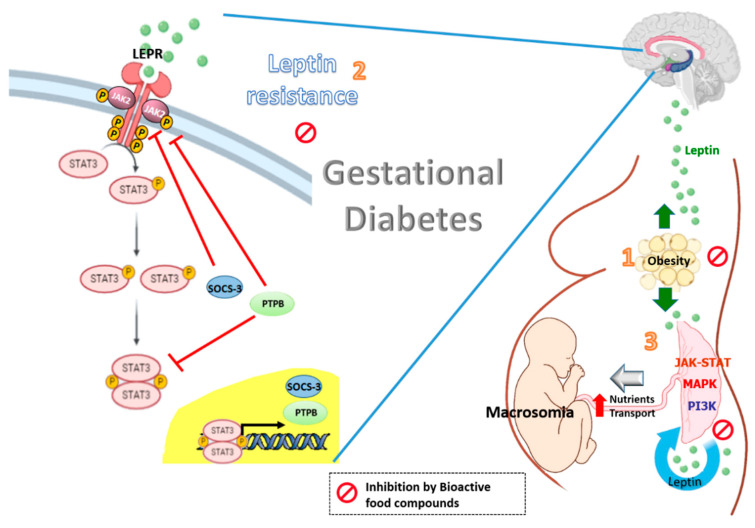
Effects of bioactive food compounds on the leptin resistance associated with obesity and gestational diabetes.

Therefore, leptin can act as metabolic switch connecting the nutritional status of the body to high energy-consuming processes. This is especially important in pregnancy, where leptin not only modulates satiety and energy homoeostasis in the mother [13,33], but it is also produced by the placenta, which responds to the environment attempting to maintain fetal viability. This placental production of leptin is one of the major sources of higher levels of maternal circulating leptin other than maternal gain of fat mass [34]. Thus, the effects of placental leptin on the mother may contribute to endocrine-mediated alterations in energy balance, such as the mobilization of maternal fat, which could further aggravate the insulin resistance associated with pregnancy and the onset of GDM [35,36]. In fact, obese pregnant women have significantly elevated plasma leptin concentrations compared with nonobese pregnant women throughout pregnancy [1]. Moreover, maternal obesity is also associated with changes in the placental function and structure, which likely impact fetal growth and development. For example, obesity has been associated with several changes related mainly with placental size, hypervascularization, higher branching capillaries of the villi (chorangiosis) [37,38] and increased glycogen deposits, among others. Increased macrophage infiltration is also evident in the placenta of obese women, suggesting an exaggeration of the inflammatory state which occurs in normal pregnancy [39]. However, it is unclear which histological changes are due to the pathophysiology and which are compensatory adaptations to this disease. Regardless, alterations in placental nutrient and hormone transporter capacity have been demonstrated in human and animal models of obesity, and are hypothesized as a mechanism leading to an accelerated fetal growth trajectory and macrosomia [1]. In this sense, we have demonstrated that the increased expression of aquaporin-9 (AQP9) (or others aquaglyceroporins) observed in placentas from obese women with GDM could be mediated by hyperleptinemia, suggesting an increase in the transport of glycerol to the fetus and thus contributing to the increased energy intake requirements in the macrosomic fetus in GDM [40]. Leptin has also been identified as a critical trophic factor that influences the development of the hypothalamic projections [40]. Alterations in the pattern of leptin secretion (premature peak, excess, or deficiency) during neonatal life could have significant adverse effects on hypothalamic development and metabolic phenotype [41] (Figure 1).

Finally, one of the peripheral functions of leptin is a regulatory role in the interplay between energy metabolism and the immune system, which is, in part, responsible for the inflammatory state associated to obesity [42]. Several inflammatory mediators produced by inflammatory cells also regulate leptin expression and promote the development of chronic inflammation [43]. In this regard, leptin effects include the inflammation and the modulation of innate and adaptive immunity [44,45]. Therefore, proinflammatory leptin actions might also have significant implications in the pathogenesis of GDM [29,46].

Taken all together, since hypothalamic inflammation results in central leptin resistance and hepatic insulin resistance [47], blocking the peripheral and central inflammation induced by a high fat diet could have the potential to treat obesity and GDM. Therefore, novel therapies incorporating effective natural agents (macro and micronutrients), particularly agents with the dual properties of preventing inflammation and controlling body weight by improving leptin sensitivity, might be an alternative intervention targeting obesity and GDM.

Leptin levels are increased in gestational diabetes with obesity (1). The high plasma leptin levels may be potentiated by leptin resistance at central level, in which SOCS3 and PTPB are induced by leptin and involving in a negative feed-back loop. The resulting effect is a decrease in the leptin-induced activation of the JAK2/STAT-3 signaling, leading to a reduction in the central effects of leptin (2). Leptin also impacts the placenta itself in an autocrine/paracrine fashion. The integration of numerous signaling by intracellular regulatory pathways such as MAPK, PI3K and JAK-STAT has been demonstrated to increase the size of the placenta and to affect placental nutrient transport and fetal growth (macrosomia) (3). Bioactive food compounds such as polyphenols might reduce circulating leptin levels, partly decreasing leptin expression in the placenta from women with GDM. The resulting effect is a decrease in the leptin resistance at a central level and optimal placental nutrients transport.

## 3. Nutrients and Bioactive Food Components Useful for Counteracting Hyperleptinemia and Leptin Resistance in GDM

Since the landmark Hyperglycemia and Adverse Pregnancy Outcomes (HAPO) study in 2010, an international consensus on diagnostic criteria has not been reached nearly a decade later [47]. However, there is now a consensus regarding the need for effective interventions targeting obesity. In this regard, since first-line medical (pharmaceutical) therapy has recently been called into question [48] and the socioemotional component of nutrition therapy for GDM has a dominant influence on adherence [49], other dietary strategies should be further investigated in detail. For example, since diet plays an important role in inflammation, and both obesity and GDM are considered a state of chronic inflammation, a healthy and active life style, which includes a diet rich in fruits, vegetables, non-sugared foods, non-ultra-processed foods associated with a higher prevention of inflammatory diseases should be incorporated into the diet [50]. However, a high consumption of red, processed meat, saturated or trans-fat, ultra-processed food based on refined ingredients or alcohol associated with pro-inflammatory processes should be avoided [35,36].

Since obese women and women with GDM show high circulating leptin levels and are hence considered leptin-resistant [51,52], we have identified nutritional strategies to counteract leptin resistance in both obesity and GDM. In this context, several micro- and macro-nutrients and bioactive food components might have the ability to increase leptin sensitivity and to reverse leptin resistance in obesity and GDM.

### 3.1. Polyphenolic Compounds

Polyphenolic compounds such as flavonoids represent an important bioactive component in plants (fruits, vegetables, legumes, tea, etc.) with some specific parts of these foods richer in flavonoids than others (for example the peel of certain fruits) [53]. The accumulation of flavonoids often occurs in plants subjected to abiotic stresses. This fact has made their determination an attractive field in food science and, in recent years, an increased number of studies have analyzed their potential benefits in human health.

As mentioned above, both the impact of nutritional status on the immune system, specially T cells [54,55] and the role of leptin as a mediator of inflammation [42], as well as a link between energy stores and the immune response, have been proposed [56,57]. In this sense, the (poly)phenols might modulate both the leptin effect and circulating leptin levels using different experimental approaches. For example, it has been reported that flavonoids have anti-inflammatory properties and thus have an important role in the control of several immune cells and immune mechanisms that are important in the inflammatory processes. More specifically, certain flavonoids (myricetin, quercetin, procyanidins) can inhibit multiple central kinases that are involved in multiple signaling pathways related to inflammation [58], such as phosphoinositol kinase, protein kinase C (PKC), phosphatidylinositol kinase and tyrosine kinase or cyclin-dependent kinase-4 [59]. Besides, flavonoids can modulate these protein kinases via inhibition of transcription factors (e.g., NF-κB and AP-1) [60]. Intriguingly, both insulin and leptin share several signaling pathways, such as mitogen-activated protein kinase (MAPK) and the phosphatidylinositol 3-kinase (PI3K) pathway, which may also activate several protein kinases involved in signal transduction during the inflammation process, via NF-κB. In this context, our group have demonstrated that the increase in placental leptin expression is mediated by NFκB signaling [61]. Therefore, in GDM, associated with insulin resistance, hyperinsulinemia and hyperleptinemia [62,63], flavonoids might downregulate the synergistic interaction between insulin and leptin signaling in the inflammatory processes. In addition, flavonoids might also decrease leptin expression in the placenta of women with GDM.

It has also been reported that high cAMP levels inhibit leptin expression by human chorionic gonadotropin (hCG), and these increased levels of cAMP have been associated with anti-inflammatory functions [64]. In this sense, flavonoids have also demonstrated the potential to block cAMP degradation and prolong cAMP signaling [65]. Finally, flavonoids may have an impact on cell activation, signaling transduction and cytokine production in several immune cells. For instance, flavonoids have been shown to inhibit maturation of dendritic cells (DCs) by suppressing the expression of CD83 and CD80, which would translate into an inhibitory effect in the secretion of pro-inflammatory cytokines [66,67]. These effects are contrary to leptin, which promotes the switch towards Th1 cell immune responses by increasing interferon-ɣ (IFN-ɣ) expression and facilitates Th17 responses. All these findings position flavonoids as modulators of immune response and, very specifically, as inhibitors of transcription factors, involved in the expression of different pro-inflammatory genes such as the leptin gene [60].

On the other hand, it is well known that the imbalance between the oxidative and anti-oxidative systems plays a crucial role in the pathogenesis of several human diseases such as obesity and diabetes, among others [68,69,70]. In this context, flavonoids are also potent antioxidants that are able to scavenge free radicals and decrease their formation. For example, grape juice by-products are a source of phenolic compounds with demonstrated antioxidant activities [71]. In these, the main phenolic compounds include flavones (luteolin) [71,72], flavonols (myricetin, fisetin, quercetin and kaempferol derivatives) [70,73], anthocyanins (cyanidin-3-glucoside) [74], flavan-3-ols (catechin and epicatechin monomers and proanthocyanidins) [75,76], stilbenes (resveratrol), and phenolic acids [74]. The most studied of these is resveratrol, which has been demonstrated to diminish circulating leptin levels and to reduce intake [77] by increasing phospho-STAT3 content in the hypothalamus, with no changes in SOCS3. This suggest that resveratrol might improve the leptin sensitivity in obesity [78,79]. Proanthocyanidins (PACs), known as condensed tannins, are found in a wide variety of fruits (e.g., berries), in addition to grapes, and other sources such as flowers, seeds of some plants, nuts or barks [80,81]. Plant and food-derived PACs are also attracting attention due to their ability to prevent chronic diseases [82]. For example, PACs from grape seeds and blackberry–blueberry fermented beverages have shown high anti-inflammatory and antioxidant activity in vitro [83] and have shown a broad therapeutic health effect against diabetes mellitus and obesity. In this regard, obesity and related complications such as GDM are linked with higher susceptibility to oxidative stress and the administration of grape seed extracts has shown an improvement in the oxidative status in obese people by inhibiting lipid peroxidation and avoiding ROS production [84]. Moreover, PACs can reduce inflammation by decreasing the oxidative stress or other indirect mechanisms [85]. In this sense, PACs from grape seed extract modulate IL-6, TNF-α and adiponectin gene expression in adipose tissue, thus, reducing the diet-induced low-grade inflammation [86]. PAC-rich extracts have also proved to be involved in obesity modulation (even at low doses) through the suppression of food intake and the increase in energy expenditure [87], possibly by mediating leptin levels. However, the mechanism underlying this effect of grape seed PACs has not been fully elucidated. Finally, grape seed extract improved the insulin resistance index as well as the plasma glucose and insulin levels in diet-induced obese animal models [88], although there are discrepancies in this regard.

Other bioactive food compounds have also been proven to be able to reduce circulating leptin levels in obesity. Myricetin, a bioflavonoid abundant in others fruits (e.g., berries), as well as tea and vegetables, has been shown to reduce hyperleptinemia and to favor insulin action via PI3-kinase pathway activation, and translocation of glucose transporter subtype 4 (GLUT4) to the cell membrane [89]. Accumulating evidence also suggests that propolis extracts (rich in flavonoids and cinnamic acid derivatives) have therapeutic effects on obesity by controlling adipogenesis, adipokine secretion, food intake, and energy expenditure. Particularly, considering the anorectic activity of leptin, propolis has potential to attenuate feeding and subsequently prevent obesity [90]. Moreover, various reports in animal and cellular models have demonstrated that propolis and its derived compounds improve insulin secretion and insulin sensitivity by modulating oxidative stress, the accumulation of advanced glycation end products (AGEs), and adipose tissue inflammation, all of which contribute to insulin resistance or defects in insulin secretion [91,92]. For example, several flavonoids in propolis, such as quercetin, chrysin, luteolin, amentoflavone, luteolin 7-O-glucoside and daidzein, have been found to have therapeutic effects in diabetic animal models by different mechanisms [93,94]. It has also been reported that propolis mitigates metabolic dysfunction through normalization of intestinal microflora [95]. Therefore, propolis intake might have beneficial effects for metabolic disorders such as GDM, attributable to flavonoids and natural phenols. However, propolis might have adverse effects on patients and, therefore, monitoring of biological effects should be carried out.

The polyphenols of olives and olive leaves also have numerous beneficial effects on human health, such as antioxidant capacity, hypoglycemic [96] and anti-inflammatory [97], as well as a coadjuvant role in the treatment of obesity [98]. In this sense, oleuropein, responsible for the bitter taste of olive leaves and drupes, and its derived form, the most abundant phenolic compounds present in olives and olive oils, are well known for their hypoglycemic property; possibly by the potential of affecting glucose-induced insulin release and/or increasing peripheral glucose uptake [99]. This hypoglycemic effect is also attributed, at least in part, to the antioxidant activity of oleuropein [100]. Moreover, it has been reported that oleuropein down-regulates leptin mRNA levels in epididymal adipose tissue and reduces serum leptin levels [101]. That is why the prophylactic use of oleuropein has been proposed in the reduction in complications resulting from oxidative stress in obesity and diabetes [99]. Other major phenolic components present—not only in olive extracts but in fruits (e.g., grapes), such as luteolin and luteolin-4′-O-β-D-glucopyranoside—have been shown to inhibit the formation of AGEs and, thus, might delay the development of diabetic complications [96]. However, these effects have been tested in animals and it is necessary to perform studies in humans in order to confirm the benefits attributed to polyphenols from olives in GDM.

### 3.2. Polyunsaturated Fatty Acids (PUFAs)

The developing fetus requires substantial amounts of fatty acids to support rapid cellular growth and activity, and especially, metabolic derivatives of the essential fatty acids such as linolenic acid (Ω-3) and linoleic acid (Ω-6) and polyunsaturated fatty acids (PUFAs) are crucial [102]. The most biologically important PUFAs are docosahexaenoic acid (DHA) and eicosapentaenoic acid (EPA) [102]. In this context, DHA appears to be crucial to the fetus and infant for early neural development (brain and visual system) [103]. However, modern dietary trends have led to an imbalance in the consumption of PUFAs, with deficiency in Ω-3 and increasing Ω-6 PUFA intake which far exceeds nutritional requirements, promoting the pathogenesis of many prevalent human diseases including GDM [104].

The placenta may play a key role in the regulation of fatty acid availability via the release of placental-derived leptin, a potent stimulator of lipolysis [105]. In fact, maternal circulating total fatty acid concentrations increase during pregnancy, enhancing placental access to fatty acids. However, as mentioned above, GDM is associated with oxidative stress and placental inflammation [106], and impaired placental fatty acid transport has been reported [107].

Given that PUFAs exhibit both anti-oxidative and anti-inflammatory activities, maternal dietary DHA and EPA supplementation has been proposed as a potential therapeutic intervention for this placenta-related disorder. For example, maternal dietary supplementation with Ω-3 PUFAs during pregnancy exerts beneficial effects such as reduced inflammation by either disrupting proinflammatory eicosanoid generation or promoting the generation of anti-inflammatory forms [108]. Moreover, it has been also reported that dietary supplementation with Ω-3 PUFAs modulates the activity of key transcription factors (peroxisome proliferator-activated receptors (PPARs) and/or nuclear factor κB (NF-κB)) and the G-protein-coupled receptor (GPR120) involved in inflammatory signaling [109,110,111]. Consequently, dietary supplementation with Ω-3 PUFAs might reduce risk of pregnancy complications [112] as well as the adipose tissue inflammation via GPR120-mediated suppression of macrophage proinflammatory cytokine secretion, including leptin [113]. Indeed, DHA and EPA have been shown to reduce circulating leptin levels activating the adenosine 5‘-monophosphate-activated protein kinase (AMPK) pathway [114]. Another beneficial effect of the Ω-3 PUFAs on GPR120 is the increase in the translocation of intracellular vesicles containing GLUT4, which enhances glucose uptake by adipocytes [111]. Oleic acid, a monounsaturated fatty acid (MUFA), also reduces hyperleptinemia via down-regulating PPARγ mRNA levels in abdominal visceral white adipose tissue in obese mice [115]. Through modulation of adipokine secretion, these fatty acids also favor insulin sensitivity [116].

Finally, excessive oxidative stress in utero-placental tissues plays a pivotal role in the development of GDM [106]. In this context, Ω-3 PUFAs could potentially limit oxidative damage by reducing ROS generation [117]. However, it would be important to ascertain the potential risks of excessive dietary PUFA intake given the susceptibility of PUFAs to lipid peroxidation, which may exacerbate cellular damage caused by an oxidative insult [118].

All together, these findings highlight the potential benefit of dietary supplementation with PUFAs to limit oxidative damage and inflammation associated with obesity and GDM, although further research in humans is required to clarify whether these fatty acids can prevent GDM and the potential risks associated if they are used as supplements.

### 3.3. Terpenes

There is evidences that cafestol and/or its metabolites (kahweol), natural diterpenes extracted from coffee beans, can prevent some chronic diseases such as metabolic disease [119,120,121]. In this context, it has been demonstrated that cafestol promotes insulin secretion and also increased glucose uptake in muscle cells, similarly to that of antidiabetic rosiglitazone. Moreover, kahweol can activate the AMPK pathway, a central modulator of the metabolism of glucose and lipid that stimulates glucose uptake and inhibits the lipid accumulation. A large number of studies have also shown that cafestol and kahweol have anti-inflammation and antioxidant activity, as well as an inhibitory effect on cell proliferation. More specifically, cafestol blocks the AP-1 pathway to reduce PGE2 production and blocks the PI3K/Akt pathway, promoting apoptosis in tumor cells [122]. All these effects could be beneficial in the placental overgrowth observed in GDM. However, despite the fact that caffeine has been shown to activate STAT-3 via decline of ER stress in the hypothalamus [123], it has been reported that kahweol down-regulated the STAT3 signaling pathway by inhibiting its constitutive phosphorylation and activation [124], which may aggravate the leptin resistance in obesity. Therefore, further research and clinical trials are needed to confirm whether the coffee diterpenes might be used to prevent or treat GDM in humans.

Evidence has been reported regarding the effect of tea preventing obesity and abnormal glucose and lipid metabolism [125]. In this sense, in addition to phenolic components, a major bioactive component of tea extract is teasaponin, a triterpene with significant anti-inflammatory properties. More specifically, teasaponin inhibits proinflammatory cytokines by suppressing NFκB signaling upstream of IKK/IκBα [125]. The anti-inflammatory effects of teasaponin have been associated with an improved glycemic status in animal models. Moreover, teasaponin decreases the expression of hypothalamic proinflammatory cytokines as well as the inflammatory signaling in the mediobasal hypothalamus [125]. This may contribute to improved leptin sensitivity and hypothalamic leptin signaling via p-STAT3. In fact, teasaponin significantly decreases the level of SOCS3, a negative regulator of central leptin signaling in the hypothalamus of high-fat diet-induced obese mice. Therefore, teasaponin has important effects in improving glucose tolerance, central leptin sensitivity, and hypothalamic leptin signaling [125], and it might be a potential candidate as therapeutic intervention for obesity and GDM.

### 3.4. Probiotics

Early reports of experimental and human studies regarding the role of gut microbiota promoting gut barrier functions and controlling inflammatory responses have attracted scientific interest. The gut microbiota is highly sensitive to the diet and may be involved in fat accumulation, favoring hydrolysis and absorption of indigestible polysaccharides and, thus, excessive storage of nutrients [126,127]. In fact, a distinctive gut microbiota composition in obesity has been reported in humans [127]. For example, a lower fiber intake has been reported to be associated with reduced gut microbiota diversity and richness, greater abundance of genus associated with type 2 diabetes mellitus [128,129], and genus with known pro-inflammatory capacity [34]. Physiological weight gain during pregnancy [130,131] also influences the gut microbiota composition in parallel with weight gain, favoring a higher number of *Bifidobacterium* spp and a lower proportion of *Staphylococcus* spp [132]. These shift in microbial composition are more pronounced in obese pregnancy and women with overweight gain during pregnancy [130,131,132,133]. Therefore, a reasonable strategy to fight GDM might be based on specific probiotics, which might counteract excessive absorption and storage of nutrients by modification of the gut microbiota composition. Probiotics in GDM might balance the effect of aberrant indigenous microbiota and normalize the increased intestinal permeability, as well as the secretion of proinflammatory mediators, including leptin. Therefore, as mentioned in a clinical trial [134], specific probiotics or probiotic foods might be used as dietary adjuncts to reduce the risk of diseases associated with aberrant gut microbiota composition, increased intestinal permeability or altered immunological or metabolic balance such as GDM. In fact, the impact of probiotics on GDM might be more pronounced in a high-risk population (e.g., obesity). Moreover, probiotics supplementation would not only affect the maternal metabolic state, but would also modulate fetal physiology and might have a long-term programming effect on child health [135,136,137]. However, current knowledge about gut microbiota and diet response in pregnancy complicated by GDM is limited and future studies that integrate genetics and clinical variables should be taken into account.

### 3.5. Others Bioactive Compounds

Lycopene is a lipophilic carotenoid which is responsible for the red color in various vegetables and fruits, and is commonly found in tomatoes [138,139]. This carotenoid is known for its antioxidant and anti-inflammatory effects [140], and has been reported to improve diseases with chronic inflammatory backgrounds such as obesity. As mentioned above, hyperleptinemia is associated with pro-inflammatory responses and with the chronic subinflammatory state observed in obesity [141]. In this context, it has been suggested that lycopene supplementation may attenuate the inflammatory response in obesity, at least in part, by minimizing hyperleptinemia [142,143]. Other bioactive compounds of many cruciferous vegetables (e.g., watercress and broccoli) are isothiocyanates (ITCs), characterized by the presence of thiol-reactive chemicals that can modify critical cysteine residues on a variety of cellular proteins [144,145,146]. As mentioned above, accumulating evidence suggests that PTP1B could be involved in the pathways leading to leptin resistance as a major negative regulator of leptin and insulin signaling. In this sense, ITCs have been found to inactivate PTP1B [147,148], which has a reactive cysteine residue at the catalytic center [147]. Particularly, phenethyl isothiocyanate (PEITC), a relatively nontoxic constituent, in addition to inhibiting cellular PTP1B activity, has been demonstrated to enhance phosphorylation of LEPRb, JAK2, and STAT3 in the hypothalamus, resulting in the stimulation of leptin signaling and significantly reduced food intake [149].

### 3.6. Micronutrients

Micronutrients include numerous minerals and vitamins derived from the diet that are essential for cellular metabolism and optimal tissue function. It has been reported that an adequate supply of micronutrients during pregnancy may significantly reduce the risk of developing disorders of pregnancy, including GDM [150]. Throughout the course of pregnancy, there may be increased risk of micronutrient deficiency in response to the requirements of the growing fetus [151]. Therefore, although a healthy diet would be the ideal way to cover the micronutrient requirements, it is possible that the physiological challenge of pregnancy might require additional nutritional support of micronutrients [152]. The majority of supplements on the market contain a wide variety of vitamins (B group vitamins, vitamins C, D, E and folate) and minerals (iron, copper, zinc, iodine, selenium [153]. However, despite the benefits of micronutrients in supporting maternal, placental, and fetal homeostasis during pregnancy [152,154], insignificant evidence and varied results have been noted upon randomized trials of supplementation [155]. Such variability may be linked to variations in specific micronutrient supplement preparations and population contexts [155]. Therefore, the possibility that micronutrients supplementation could prevent complications of pregnancy warrants further investigation with larger trials in this field [151].

## 4. Mediterranean Diet

**Figure 2:**
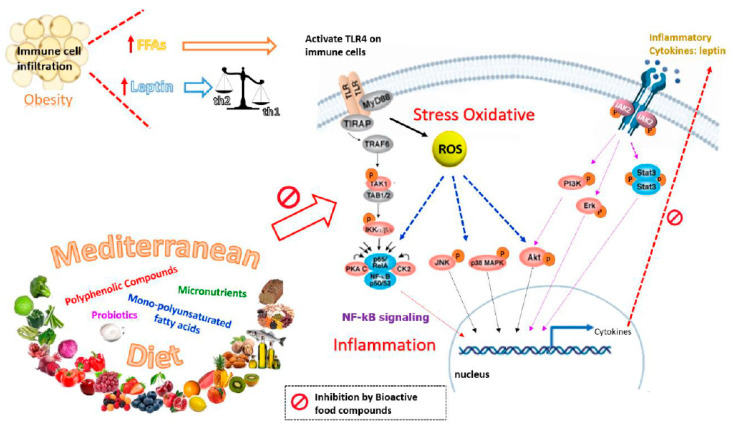
Effects of the Mediterranean diet on the oxidative stress and inflammation associated with obesity and gestational diabetes. Obesity and GDM are linked with higher susceptibility to oxidative stress and inflammation. Adipocyte hypertrophy results in elevated circulation of free fatty acids (FFAs) and increased secretion of leptin, which drives T cells toward a pro-inflammatory phenotype (Th1). These in turn result in immune cell infiltration and the activation of pro-inflammatory signaling pathways. Bioactive food compounds in the Mediterranean diet such as polyphenols exert their anti-inflammatory activity by inhibiting ROS production and inhibiting multiple central kinases that are involved in multiple signaling pathways related to inflammation, such as NF-κB, MAPKs and PI3/Akt signaling pathways.

Despite the publication of numerous randomized trials on diet and lifestyle interventions in pregnancy [156], as mentioned above, no clear dietary recommendations have emerged to improve pregnancy outcomes for women with metabolic risk factors, particularly GDM. This can be attributed to the lack of robust evidence on effectiveness of the diet [157]. The traditional diet, “Mediterranean diet”, has long been associated with preventive activity against chronic inflammation-associated diseases, which are supported by observational and epidemiological data. The Mediterranean-style diet includes components such as a high intake of nuts, extra virgin olive oil, fruit, vegetables, non-refined grains, legumes and micronutrients, as well as moderate to high consumption of fish and low consumption of processed meat, sugary drinks, fast food, and food rich in animal fat [158]. These key components of this diet might help to control the activity of obesity and GDM as well as other inflammatory pathologies. In fact, the Mediterranean diet has been recently found to reduce the risk of GDM in a multicenter randomized trial [159]. It is possible that this beneficial effect on GDM could be due to the high intake of dietary polyphenols and micronutrients found in key components of the Mediterranean diet, such as extra virgin olive oil, grapes and nuts, which together activate insulin receptors, increase the uptake of glucose in the insulin-sensitive tissues, stimulate insulin secretion, and reduce insulin and leptin resistance. However, it should be stressed that the Mediterranean diet is a complex matrix of compounds and its biological activity cannot be attributable only to polyphenols and micronutrients. In fact, it is very likely that compounds of this diet could have not only additive, but also synergic or complementary activities to phenolic components. With the growing incidence of obesity and GDM, health effects of the Mediterranean diet and its multiple bioactive components will be of relevance not only in the treatment of obesity and GDM but, perhaps more importantly, also in their prevention. Therefore, a simple, individualized, Mediterranean-style diet in pregnancy could have the potential to reduce gestational weight gain and the risk of GDM. Figure 2 summarizes the 442 preventive effects of nutrients from the Mediterranean diet on leptin resistance.

## 5. Conclusions

The high social and economic impact of the growing incidence of obesity and GDM have strongly motivated original investigations and the search for novel and rational preventive strategies. Data reported in the literature, and gathered in this review, show that there is scientific evidence supporting the anti-obesity effect of several bioactive compounds present in the Mediterranean diet. In parallel with the reduction in body fat accumulation, other features which are typical of pregnancy with obesity, such as GDM, increased leptin and insulin resistance, stress oxidative and low-grade inflammation, are also improved by these bioactive compounds. Some of the mechanisms of action underlying these effects have been revealed by preclinical studies. Particularly, the leptin sensitivity effect of polyphenolic compounds (one of the most interesting group of bioactive compounds), as well as the improvement of several comorbidities observed in GDM, has also been detailed. However, despite the publication of numerous studies on diet and lifestyle interventions in pregnancy, no clear dietary recommendations have emerged to improve pregnancy outcomes for women with metabolic risk factors. In this regard, observational evidence on the Mediterranean-style diet intervention in pregnancy and potential reductions in weight gain and the risk of gestational diabetes should be taken into account. The key components of this diet include, at the very least, a high intake of polyphenolic and other bioactive compounds. It addresses some important benefits by using additional non-pharmacological therapy that is based on natural compounds and, moreover, it would be feasible to implement in pregnant women. It should be pointed out that future studies should investigate which bioactive compounds present in the Mediterranean diet are responsible for their effects, as well as the potential synergies between them. This strategy would be helpful to find new therapeutic interventions to prevent or treat GDM.
